# Baiting blast fungi: an engineered NLR domain improves effector recognition

**DOI:** 10.1093/plcell/koag189

**Published:** 2026-06-18

**Authors:** Jan W Huebbers

**Affiliations:** Assistant Features Editor, the Plant Cell, American Society of Plant Biologists; Unit of Plant Molecular Cell Biology, Institute for Biology I, RWTH Aachen University, Worringerweg 1, Aachen 52056, Germany

Plant pathogens such as bacteria, oomycetes, and fungi secrete effector proteins to weaken the plant immune system and promote susceptibility. These effectors enter host cells, where they target host proteins. In response, plants have evolved molecular sensors known as nucleotide-binding leucine-rich repeat (NLR) receptors to recognize pathogen effectors. Some NLRs use particularly sophisticated strategies for effector recognition. Like bait on a fishing hook, certain NLRs carry integrated domains that mimic the effector's usual host target. If the effector takes the bait, the receptor itself, or an associated helper NLR, triggers immune responses that may culminate in induced cell death, depriving pathogens of the living host tissue they need to thrive.

Effectors that are recognized by corresponding plant immune receptors and thereby confer avirulence are typically denoted with the prefix “AVR.” For example, the fungus *Magnaporthe oryzae* is the causal agent of the rice blast and wheat blast diseases and secretes the effector AVR-Pii. In rice (*Oryza sativa*), AVR-Pii is recognized by an NLR receptor. However, its original targets are the rice Exo70 proteins *Os*Exo70F2 and -F3 (Fujisaki et al. 2015). Exo70 proteins are subunits of a molecular docking station that mediates the contact between secretory vesicles and the plasma membrane. Compared to animals and fungi, the *Exo70* gene family has considerably expanded in plants. As some Exo70 isoforms have acquired roles in immunity, they became targets for pathogen effectors.

Recently, the barley (*Hordeum vulgare*) sensor NLR *Hv*RGH2 was found to carry an integrated Exo70 domain (Brabham et al. 2024), suggesting that *Hv*RGH2 and its helper NLR *Hv*RGH3 function in immunity against Exo70-targeting effectors. The main effector recognized by *Hv*RGH2 is still elusive. However, the integrated Exo70 domain provides an intriguing parallel to AVR-Pii recognition. In new work, **Indira Saado and colleagues** (Saado et al. 2026) show that AVR-Pii from rice blast and wheat blast bind to the Exo70 domain of *Hv*RGH2. Using structure-guided engineering, they developed an RGH2^+^ variant that elicited stronger immune responses than the native RGH2 variant.

The authors first carried out de novo transcriptome assembly for 102 Poales species, an order of monocotyledonous flowering plants that includes grasses. They identified 184,775 potential NLRs, 82 of which contained an Exo70 integrated domain. Six of these integrated domains were confidently mapped onto the Exo70 gene family, and phylogenetic analysis associated them with the Exo70F and Exo70FX clades. The authors tested 5 of these integrated domains for interaction with AVR-Pii variants. Among these, only RGH2-Exo70 interacted with some of the tested effectors, including AVR-Pii from rice and wheat blast isolates.

Interestingly, the authors observed that the Exo70 integrated domain from RGH2 showed weaker interaction with AVR-Pii variants than their *Os*Exo70F3 positive control. Based on the *Os*Exo70F2/AVR-Pii crystal structure (De la Concepcion et al. 2022) and sequence similarity to the AVR-Pii binding site in RGH2, they engineered RGH2^+^. RGH2^+^ carries 3 mutations in the AVR-Pii binding site compared with RGH2 (Fig. 1) and binds AVR-Pii variants from rice and wheat blast more strongly than RGH2, although not as strongly as *Os*Exo70F3. To test whether improved effector binding also translates into stronger immune responses, the authors co-expressed genes encoding RGH2 or RGH2^+^, its helper NLR RGH3, and AVR-Pii in *Nicotiana benthamiana*. RGH2^+^ elicited qualitatively stronger cell death than RGH2, and the wheat blast AVR-Pii variant induced stronger cell death than the rice blast variant. The authors then generated transgenic barley lines accumulating RGH2/RGH3 or RGH2^+^/RGH3. Strikingly, only the latter exhibited reduced susceptibility to an *M. oryzae* isolate that produced the wheat blast AVR-Pii variant.

**Figure 1:**
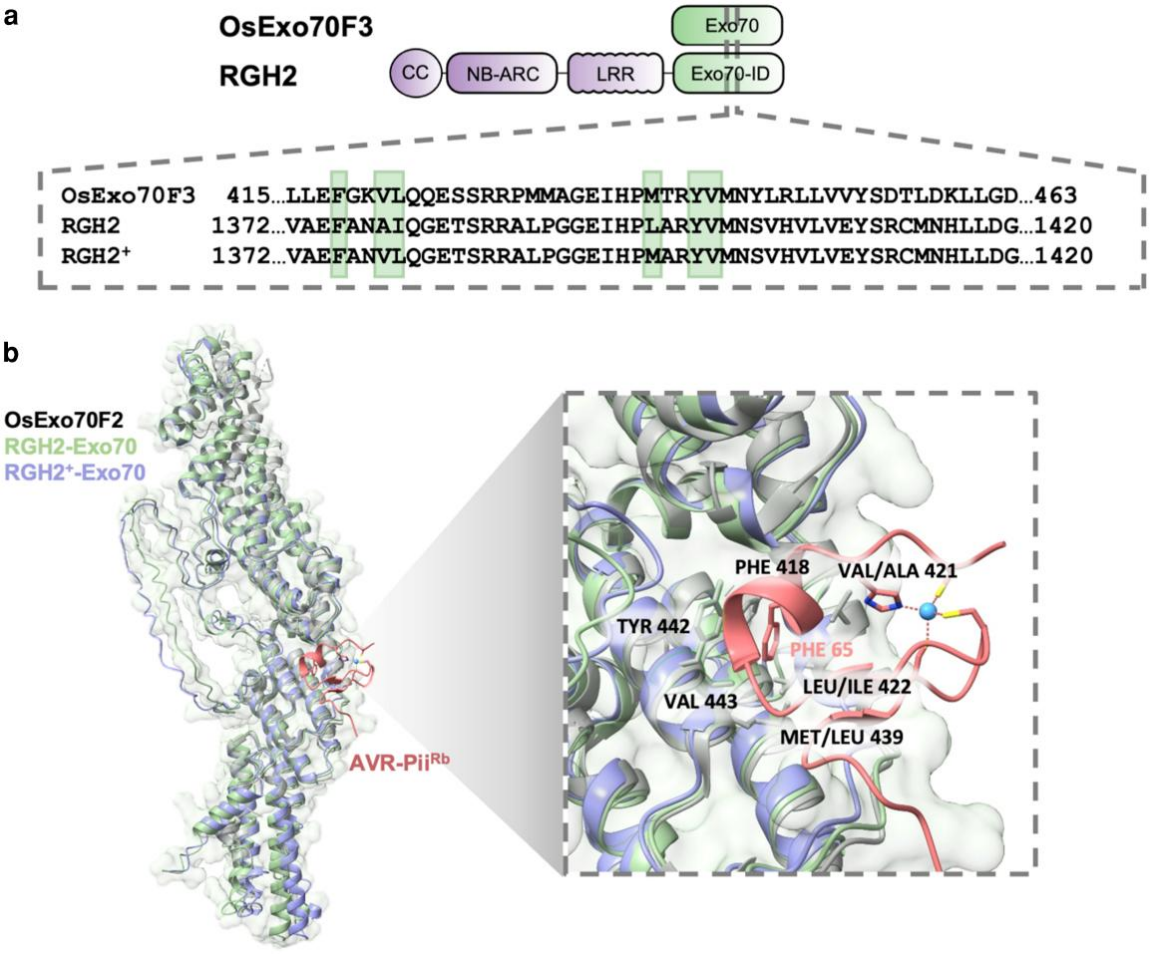
Engineered RGH2^+^ carries 3 mutations for improved AVR-Pii binding. a) Sequence alignment of the AVR-Pii binding interface in *Os*Exo70F3 and RGH2/RGH2^+^. Residues that establish effector binding are highlighted. b) Superposition of the experimental *Os*Exo70F2/AVR-Pii complex and structure predictions for RGH2-Exo70 and RGH2^+^-Exo70. The inset shows the effector-binding interface, with the rice blast AVR-Pii shown in peach. Sequence alignment and structural superposition have been retrieved from **Saado and coworkers** (2026).

Engineering plant immune receptors has emerged as a valuable tool for plant protection. Here, the authors show that such strategies can accelerate the breeding of resistant cultivars, for example by tailoring the specificity of effector recognition. While experimental structures such as the *Os*Exo70F2/AVR-Pii complex provide valuable templates, structure-guided engineering also greatly benefits from AI-based tools for structure prediction and pipelines for de novo protein design.

## Recent related articles in *The Plant Cell*

- Bentham et al. (2023) show that compatible combinations of rice Pik sensor and helper NLRs variants potentially facilitate the engineering of new effector recognition specificities without triggering receptor autoactivity.
- Huebbers et al. (2024) show that EXO70 and MLO proteins interact isoform-specifically to modulate trichome cell wall composition and powdery mildew susceptibility in *Arabidopsis*.
- Liu et al. (2024) show that EXO70B1-mediated autoimmunity in *Arabidopsis* depends on a TN2–CPK5–CAMTA3 signaling module, in which CPK5 phosphorylates and destabilizes the CAMTA3 transcriptional repressor to activate immune responses.
- Sutherland et al. (2025) highlight how natural diversity at the DNA, RNA, and protein levels can inform the engineering of plant immune receptors from *Arabidopsis* to crop plants.

## Data Availability

No new data were generated or analyzed in support of this article.
